# DARS-AS1 modulates cell proliferation and migration of gastric cancer cells by regulating miR-330-3p/NAT10 axis

**DOI:** 10.1515/med-2022-0583

**Published:** 2022-12-13

**Authors:** Chunjuan Du, Xia Han, Yanyan Zhang, Fengli Guo, Haibin Yuan, Feng Wang, Mianli Li, Fangling Ning, Weibo Wang

**Affiliations:** Department of Oncology, Shandong Provincial Hospital Affiliated to Shandong University, Jinan, Shandong, 250021, China; Department of Oncology, Binzhou Medical University Hospital, Binzhou, Shandong, 256603, China; Department of Pediatrics, Binzhou Medical University Hospital, Binzhou, Shandong, 256603, China; Department of Breast Surgery, Binzhou Medical University Hospital, Binzhou, Shandong, 256603, China; Department of Health Management, Binzhou Medical University Hospital, Binzhou, Shandong, 256603, China; Department of Oncology, Shandong Provincial Hospital Affiliated to Shandong University, No 324, Jingwuweiqi Road, Jinan, Shandong, 250021, China

**Keywords:** gastric cancer, DARS-AS1, NAT10, miR-330-3p, progression

## Abstract

The long noncoding RNA DARS-AS1 was aberrantly expressed and participated in several human cancer progressions, whereas whether DARS-AS1 is involved in human gastric cancer remains unclear. This study aimed to investigate the influence of DARS-AS1 on gastric cancer progression and explore the potential regulatory network of DARS-AS1/miR-330-3p/NAT10. The expression levels of DARS-AS1, miR-330-3p, and NAT10 were measured by quantitative real-time polymerase chain reaction. The CCK-8 assay and Transwell assay were used to determine the cell viability, migration, and invasion capacities, respectively. The target association between miR-330-3p and DARS-AS1 or NAT10 was confirmed using a luciferase reporter assay. In result, DARS-AS1 levels were elevated in tumor tissues and associated with shorter overall survival in patients with gastric cancer. Knockdown of DARS-AS1 could hamper cell viability, migration, and invasion in gastric cancer cells. DARS-AS1 acts as a competitive endogenous RNA to regulate the NAT10 expression by sponging miR-330-3p in gastric cancer cells. In conclusion, DARS-AS1 was elevated in gastric cancer, and DARS-AS1/miR-330-3p/NAT10 signaling offered some new horizons for predicting prognosis and a novel therapeutic method for the treatment of gastric cancer.

## 1 Introduction

Gastric cancer is a prominent public health problem, which is the gastrointestinal malignant tumor in the digestive system with the highest morbidity and mortality [1]. The pathogenesis of gastric cancer is a complex process involving interactions among environmental, host, and bacterial factors, leading to different molecular changes at the genetic and epigenetic levels [2]. Gastric cancer has the characteristics of insidious onset and insignificant early symptoms; thus, the disease would have already entered the advanced stage when most patients are diagnosed with the disease, accompanied by lymph node metastasis or distant organ metastasis, and hence the prevention and treatment of gastric cancer became difficult [3]. Although available therapies are constantly being updated, there is little effect on improving the survival of patients, and hence the research on the molecular mechanism of the occurrence and progression of gastric cancer must be strengthened and potential ideal therapeutic targets screened.

Long noncoding RNAs (lncRNAs), a type of noncoding RNAs (more than 200 nucleotides in length), have pivotal roles in the prognosis and progression of cancers by functioning as competitive endogenous RNA (ceRNA) of microRNA (miRNA) in regulating gene expression [4,5]. In recent years, maladjusted lncRNAs are involved in various pathophysiological processes of cancers, including cell viability, migration, apoptosis, and invasion [6,7]. LncRNA aspartyl-tRNA synthetase anti-sense 1 (DARS-AS1) is upregulated and plays a promoting role in regulating the growth and metastasis of cancers, such as lung cancer [8], hepatocellular carcinoma [9], and ovarian cancer [10]. However, the biological function of DARS-AS1 in gastric cancer remains elusive. DARS-AS1 could affect tumorigenesis by modulating miRNAs, such as miR-532-3p [11], miR-628-5p [12], and miR-194-5p [13]. miR-330-3p was dysregulated in various cancers [14,15,16]. For instance, miR-330-3p was downregulated in gastric cancer tissues, cell lines, and serum samples [17,18]. Human N-acetyltransferase 10 (*NAT10*) is one of the targets of miR-330-3p, which is highly expressed and may be a pro-oncogene in several tumors, including hepatocellular carcinoma and gastric cancer [19,20]. Bioinformatics analysis shows that DARS-AS1/miR-330-3p/NAT10 interaction may be involved in the tumorigenesis of gastric cancer.

Here, the expression of DARS-AS1 in gastric cancer tissues and cells was determined. The clinical significance of DARS-AS1 was also evaluated. The impacts of DARS-AS1 on cell viability, migration, and invasion were explored via functional experiments. LncRNAs could function as ceRNA to regulate gene expression at the transcriptional or post-transcriptional level by sponging miRNAs. In this study, the molecular mechanism underlying DARS-AS1/miR-330-3p/NAT10-regulated gastric cancer was probed.

## 2 Materials and methods

### 2.1 Tissue specimens and cell culture

Tumor tissues and adjacent non-tumor tissues of 113 gastric cancer patients (average age 56.47 ± 10.78 years old) were collected in Binzhou Medical University Hospital from January 2014 to November 2016. All tissue specimens were made anonymous and stored in liquid nitrogen for the following experiments. Major inclusion criteria were as follows: (1) patients who were all histologically diagnosed with gastric cancer; (2) patients with no other malignant tumors or other severe diseases in other organs; (3) patients who had not received anti-tumor therapies; and (4) patients with complete clinical characteristic information. After surgery, a 5-year follow-up survey was carried out to collect the overall survival information. The current study and the experiments were approved based on the protocol of the Ethics Committee of Binzhou Medical University Hospital. The written informed consents were signed before surgical resection by the patients.

Six gastric cancer cell lines (AGS, KATO-III, HGC-27, NCI-N87, MKN45, and SNU-1) and one human gastric mucosal epithelial cell line GES-1 were obtained from the Shanghai Institute of Biochemistry and Cell Biology (Shanghai, China). The cells were cultured in a Dulbecco’s Modified Eagle’s Medium with 10% fetal bovine serum (FBS; Invitrogen) and then incubated in an incubator with 5% CO_2_ at 37°C for further experiments.

### 2.2 Cell transfection

shRNA was used to downregulate the DARS-AS1 expression or NAT10 expression (RiboBio, Guangdong, China). Scrambled shRNA was transfected into gastric cancer cells as a negative control (shRNA-NC). pcDNA3.1-DARS-AS1 (oe-DARS-AS1) was used to upregulate DARS-AS1 expression, and the pcDNA3.1 vector was used as a negative control. miR-330-3p mimic, mimic NC, miR-330-3p inhibitor, or inhibitor NC (inhi-NC) was used to regulate the miR-330-3p expression (RiboBio). Then, transfection was carried out using Lipofectamine 2000 (Invitrogen).

### 2.3 RT-qPCR assay

To investigate differential ncRNAs and gene expression in gastric cancer tissues and cell lines, total RNA was isolated using Trizol reagent (Invitrogen) and quantified using the NanoDrop-1000 spectrophotometer (NanoDrop Technologies; Thermo Fisher Scientific, USA). Then, total RNA was reverse transcribed to cDNA using the PrimeScript RT reagent kit (TaKaRa, Shiga, Japan) or TaqMan MicroRNA Reverse Transcription Kit (Thermo Fisher Scientific) and stored at −20°C for subsequent RT-qPCR. RT-qPCR analysis was performed in triplicate using SYBR Premix Ex Taq (TaKaRa, Japan) for lncRNA and NAT10 or an all-in-one miRNA RT-qPCR Detection kit (GeneCopoeia, Inc.) for miRNA on a 7900 HT Real-Time PCR System (Applied Biosystems, Foster City, USA) to determine the expression of DARS-AS1 or mRNA expression levels of *NAT10*. The expression levels were calculated using the 2^−ΔΔCt^ equation. Vinculin was applied as internal references for mRNA and U6 for miRNA.

### 2.4 CCK-8 assay

Cell proliferation was analyzed using the Cell Counting Kit-8 (CCK-8, Dojindo, Japan). After transfection, 1,000 gastric cancer cells were seeded in 96-well plates and cultured in a culture medium with 10% FBS. The CCK-8 was joined to the plates at 0, 24, 48, 72, and 96 h and cultured for a further 2 h. The spectrophotometer (Multiskan MK3; Thermo Fisher, Waltham, USA) was used to measure the absorbance at 450 nm.

### 2.5 Transwell migration and invasion assay

The effects on cell invasive capacities or migratory abilities were explored using a 24-well Transwell chamber (Corning, NY, USA) precoated with Matrigel (BD Biosciences, San Jose, CA, USA) or without Matrigel, respectively. Briefly, 5 × 10^4^ AGS and MKN45 cells suspended in serum-free medium were added on the upper chambers and the medium with 10% FBS was added on the bottom chambers as an attractant. After 24 h of cell culture, migratory or invasive cells on the bottom were stained with crystal violet and counted under a light microscope from five randomly selected fields.

### 2.6 Dual-luciferase reporter assay

The fragment from 3′-UTR of DARS-AS1 including miR-330-3p binding sites was cloned into the pmirGLO vector (Promega, Madison, WI, USA) to form the reporter vector DARS-AS1-WT. The mutant vector (DARS-AS1-MUT) was formed by mutating miR-330-3p binding sites. Similarly, NAT10-WT and NAT10-MUT vectors were also formed using the pmirGLO luciferase vector. Then, the vectors and miR-330-3p mimic, inhibitor, or NCs were cotransfected into MKN45 cells with the help of Lipofectamine 2000 (Invitrogen). The luciferase activities were measured using the Dual-luciferase Reporter Assay System (Promega).

### 2.7 Statistical analysis

All the experiments were performed in triple and repeated at least three times, and the data were presented as mean ± standard deviation. The statistical comparisons were evaluated by paired Student’s *t*-test or one-way analysis of variance. The Kaplan–Meier curve method was used to estimate the survival curve, and multivariate Cox regression analysis was used to explore the prognostic risk factors. All the statistical comparisons were performed using SPSS 20.0 software (SPSS Inc., Chicago, IL, USA) and GraphPad 7.0 (GraphPad Software, La Jolla, USA), and statistical significance was defined as *P* < 0.05.

## 3 Results

### 3.1 DARS-AS1 was upregulated in gastric cancer and associated with overall survival

**Figure 1:**
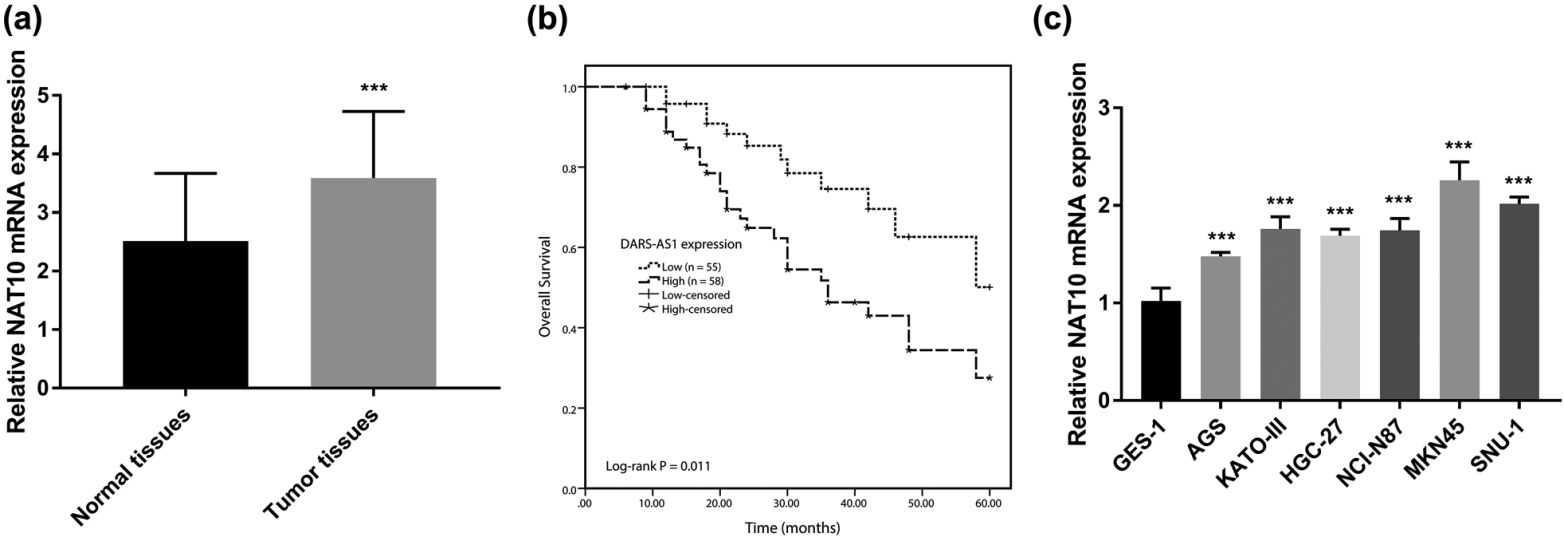
DARS-AS1 was upregulated in gastric cancer and associated with the overall survival of patients. (a) Relative expression of DARS-AS1 in gastric cancer tissues and adjacent normal tissues (*n* = 113). ***P < 0.001. (b) Overall survival analysis of the correlation between survival rate and DARS-AS1 levels in patients with gastric cancer. (log-rank test *P* = 0.011). (c) The levels of DARS-AS1 in several gastric cancer cells. **P < 0.01, ***P < 0.001.

First, DARS-AS1 expression was detected using RT-qPCR in gastric cancer tissues and corresponding adjacent tissues. As displayed in Figure 1a, the DARS-AS1 levels were significantly higher in 113 cases of cancer tissues compared with corresponding normal tissues (*P* < 0.001). A total of 113 gastric cancer patients were divided into groups according to the mean expression of DARS-AS1 in tumor tissues: a low DARS-AS1 expression group (below the mean DARS-AS1 expression) and a high DARS-AS1 expression group (above the mean DARS-AS1 expression). The clinical analysis using the *χ* ^2^ test (Table 1) showed that patients with high TNM stage or lymph node metastasis have higher DARS-AS1 expression, suggesting that DARS-AS1 expression correlates with T category (*P* = 0.040), N category (*P* = 0.016), and TNM stage (*P* = 0.005).

**Table 1:** Correlation between DARS-AS1 expression and clinical characteristics in gastric cancer patients

Parameters	Cases (*n* = 113) (%)	DARS-AS1 expression		*P* values
		Low (*n* = 55) (%)	High (*n* = 58) (%)	
Age				0.621
<60	61 (53.98)	31 (56.36)	30 (51.72)	
≥60	52 (46.02)	24 (43.64)	28 (48.28)	
Sex				0.319
Female	44 (38.94)	24 (43.64)	20 (34.48)	
Male	69 (61.06)	31 (56.36)	38 (65.52)	
Location				0.155
Cardia	54 (47.79)	22 (40.00)	32 (55.17)	
Antrum	25 (22.12)	16 (29.09)	9 (15.52)	
Body	34 (30.09)	17 (30.91)	17 (29.31)	
T category				0.040
T1 + T2	78 (69.03)	43 (78.18)	35 (60.34)	
T3 + T4	35 (30.97)	12 (21.82)	23 (39.66)	
Differentiation				0.585
Well moderate	69 (61.06)	35 (63.64)	30 (51.72)	
Poor	44 (38.94)	20 (36.36)	24 (41.38)	
N category				0.016
N0	76 (67.26)	43 (78.18)	33 (56.90)	
N1–N3	37 (32.74)	12 (21.82)	25 (43.10)	
TNM stage				0.005
I–II	88 (77.88)	49 (89.09)	39 (67.24)	
III	25 (22.12)	6 (10.91)	19 (32.76)	

The study result of DARS-AS1 expression and prognosis in patients with gastric cancer suggests that DARS-AS1 expression is related to the prognosis of patients. The overall survival of gastric cancer patients with high DARS-AS1 expression was significantly shorter than that of patients with low DARS-AS1 expression (log-rank test *P* = 0.011, Figure 1b). Moreover, multivariate Cox regression analysis (Table 2) revealed that, similar to N category (HR = 2.363, 95% CI: 1.115–5.009, *P* = 0.025) and TNM stage (HR = 2.176, 95% CI: 1.122–4.219, *P* = 0.021), DARS-AS1 expression was associated with overall survival as an independent prognostic factor (HR = 2.729, 95% CI: 1.263–5.898, *P* = 0.011). These data revealed that DARS-AS1 might be a novel prognostic predictor for gastric cancer.

**Table 2:** Multivariate Cox regression analysis of independent factors influencing overall survival

Parameters	Multivariate Cox analysis		
	HR	95% CI	*P* values
DARS-AS1 expression	2.729	1.263–5.898	0.011
Age	1.289	0.635–2.618	0.483
Sex	1.668	0.727–3.825	0.227
Location	1.426	0.616–3.305	0.407
T category	2.153	0.938–4.946	0.071
Differentiation	1.650	0.777–3.505	0.192
N category	2.363	1.115–5.009	0.025
TNM stage	2.176	1.122–4.219	0.021

Furthermore, the DARS-AS1 was also markedly upregulated in gastric cancer cell lines compared with gastric mucosal epithelial cell line GES-1 (*P* < 0.01, Figure 1c). Thus, these data confirmed that DARS-AS1 expression was elevated in gastric cancer tissues and cell lines, which was related to poor survival outcomes. Meanwhile, AGS and MKN45 cells were used in subsequent experiments.

### 3.2 Influence of DARS-AS1 knockdown on cell viability and invasion

**Figure 2:**
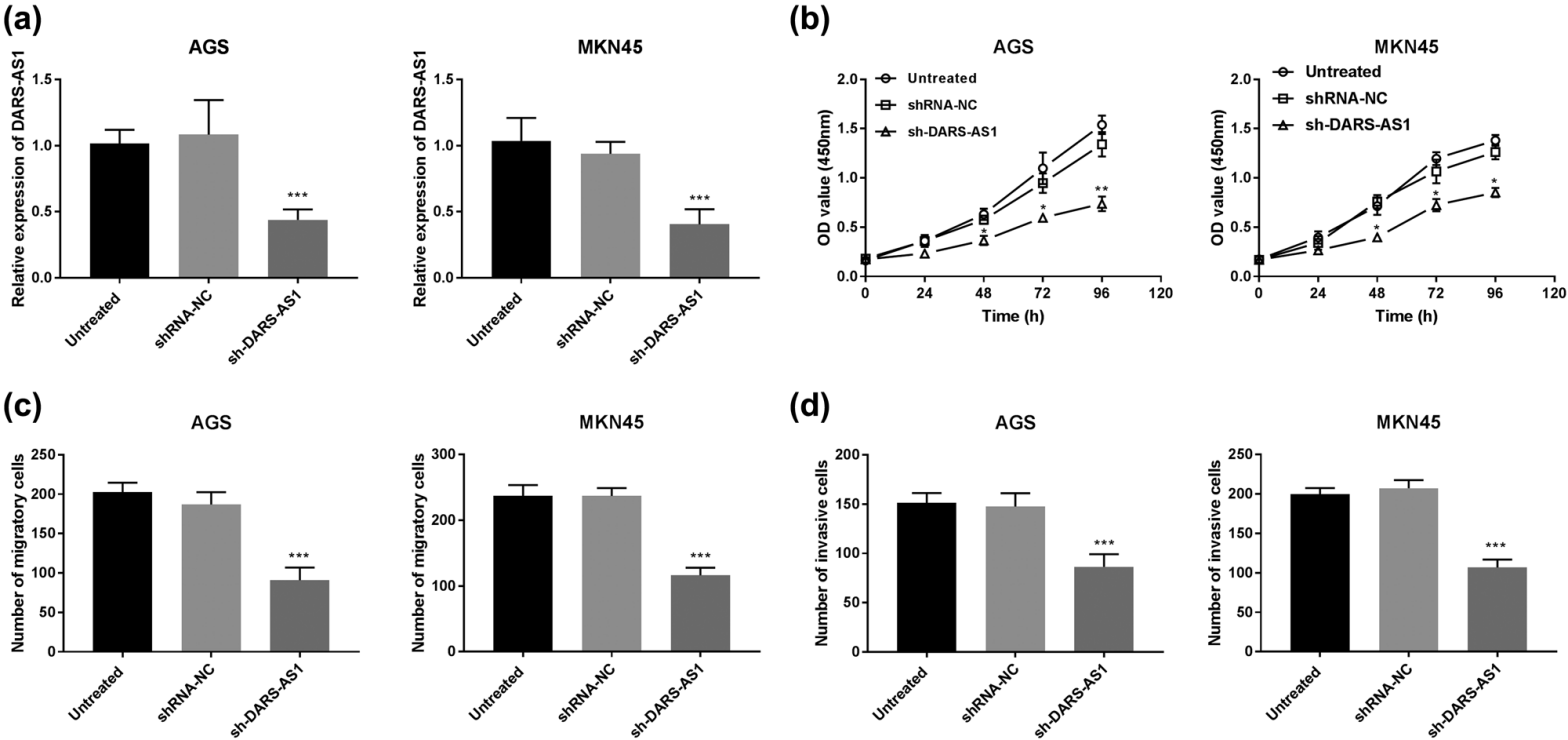
Downregulation of DARS-AS1 suppresses proliferation, migration, and invasion of AGS and MKN45 cells. (a) AGS and MKN45 cells transfected with DARS-AS1 shRNA or shRNA NC were analyzed using RT-qPCR. (b) Cell viability was analyzed using CCK-8 assay in gastric cancer cells transfected with sh-DARS-AS1. (c) Cell migration abilities were detected using Transwell migration assay. (d) The invasive capacities of AGS and MKN45 were measured by Transwell invasion assay. ***P < 0.001.

Next, the effect of DARS-AS1 on gastric cancer cellular behaviors was determined in gastric cancer cells. The knockdown efficiency of sh-DARS-AS1 was confirmed in AGS and MKN45 cells after transfection. The results uncovered that sh-DARS-AS1 was effective to downregulate the DARS-AS1 expression in AGS and MKN45 cells (*P* < 0.001, Figure 2a). The CCK-8 assay results indicated that the silencing of DARS-AS1 notably repressed cell viability of AGS and MKN45 cells (*P* < 0.05, Figure 2b). Simultaneously, the reduced expression of DARS-AS1 subdued the migratory and invasion abilities of AGS and MKN45 cells (*P* < 0.001, Figure 2c and d). These results implied that DARS-AS1 may play an oncogenic role in gastric cancer.

### 3.3 DARS-AS1 upregulates NAT10 expression by competitively binding to miR-330-3p

**Figure 3:**
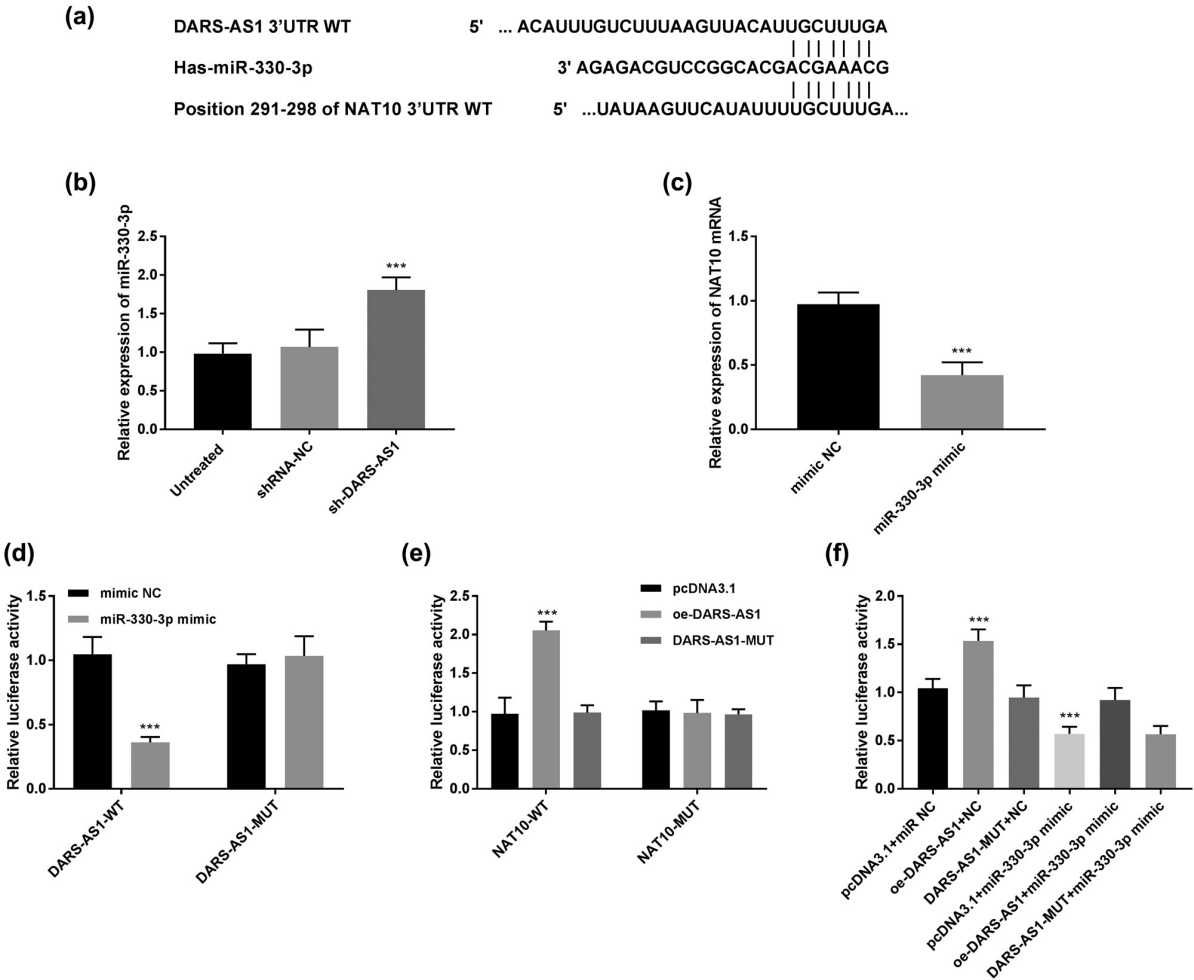
miR-330-3p was a target of DARS-AS1, and NAT10 was a downstream target of miR-330-3p. (a) The potential binding sites of miR-330-3p on lncRNA DARS-AS1 and NAT10. (b) Downregulation of DARS-AS1 by sh-DARS-AS1 increased the miR-330-3p levels in MKN45 cells. ***P < 0.001. (c) Increased expression of miR-330-3p by miR-330-3p mimic restrained the NAT10 mRNA levels. ***P < 0.001. (d) The luciferase activities of DARS-AS1-WT were inhibited by upregulation of miR-330-3p. ***P < 0.001. (e) Overexpression of DARS-AS1 increased luciferase activity of NAT10 WT-3’UTR. ***P < 0.001. (f) Transfection with miR-330-3p mimic eliminated the increased luciferase activity of NAT10 WT-3′-UTR induced by overexpression of DARS-AS1. ***P < 0.001.

LncRNA–miRNA–mRNA interactions are associated with many biological processes. It is found that DARS-AS1 has predicted targeting sites of miR-330-3p (DIANA: https://diana.e-ce.uth.gr/home) and miR-330-3p has binding sites with NAT10 (TargetScan: http://www.targetscan.org/vert_72/ and miRDB: http://mirdb.org/) (Figure 3a). We speculated that DARS-AS1 may act as a ceRNA by sponging miR-330-3p to regulate NAT10 expression during gastric cancer progression. The miR-330-3p levels were first determined in MKN45 cells with transfection of sh-DARS-AS1. The results in Figure 3b exhibited that the knockdown of DARS-AS1 increased miR-330-3p expression (*P* < 0.001). Interestingly, the expression of NAT10 mRNA was decreased by miR-330-3p mimic (*P* < 0.001, Figure 3c). Furthermore, dual-luciferase reporter assays were performed to verify the relationship between miR-330-3p and DARS-AS1 or NAT10. It is observed that increased expression of miR-330-3p decreased the luciferase activities of DARS-AS1-WT, whereas no effect on the DARS-AS1-MUT was observed (*P* < 0.001, Figure 3d). In addition, the luciferase activities of NAT10-WT were increased by DARS-AS1 overexpression, but DARS-AS1-MUT overexpression did not affect the luciferase activities (*P* < 0.001, Figure 3e). Importantly, increased expression of miR-330-3p smoothed the increased luciferase activity of NAT10-WT induced by DARS-AS1 (Figure 3f). Taken together, DARS-AS1 may undertake a ceRNA of miR-330-3p.

### 3.4 Decreased expression of DARS-AS1 weakened cellular activities of gastric cancer cells via miR-330-3p/NAT10 axis

**Figure 4:**
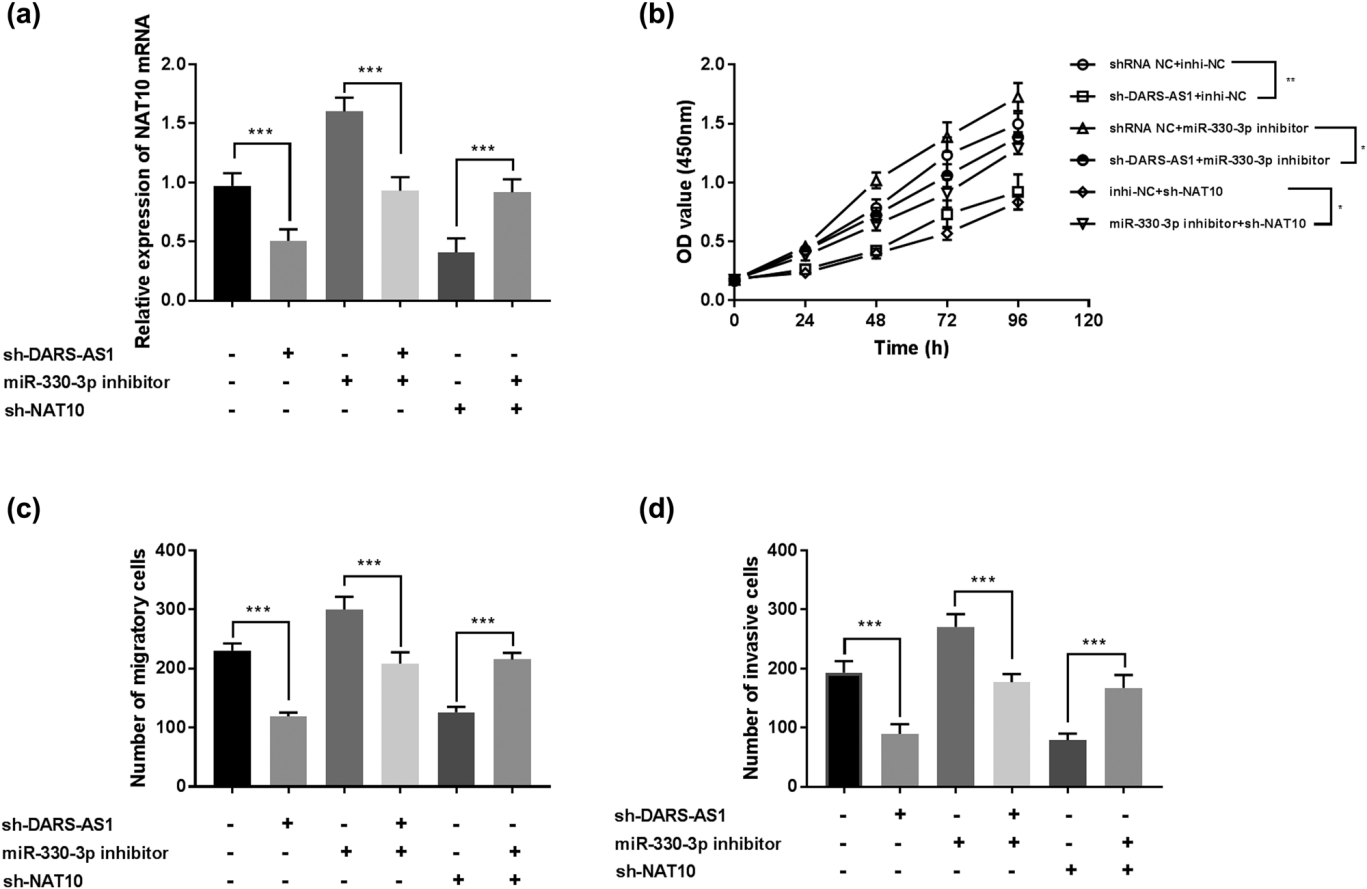
Effect of DSRS-AS1/miR-330-3p/NAT10 on cellular activities in MKN45 cells. (a) The expression of NAT10 mRNA levels was detected using RT-qPCR in MKN45 cells. ***P < 0.001. (b) The CCK-8 experiment was applied to determine cell viability in MAK45 cells. *P < 0.05, **P < 0.01. (c) Cell migration abilities of tumor cells were assessed by Transwell migration assay. ***P < 0.001. (d) The invasive capacities were assessed by Transwell invasion assay. ***P < 0.001.

The molecular mechanism of DARS-AS1 in gastric cancer was probed using MKN45 cells. The transfection efficiency was detected using RT-qPCR, and the results manifested that either downregulation of DARS-AS1 or silencing of NAT10 decreased the expression of NAT10 mRNA, while this effect was reversed by decreased expression of miR-330-3p (*P* < 0.001, Figure 4a). Then, CCK-8 assay and Transwell assays displayed that the silencing of DARS-AS1 or NAT10 alleviated proliferation (*P* < 0.05, Figure 4b), migration (*P* < 0.001, Figure 4c), and invasion (*P* < 0.001, Figure 4d) capacities of MKN45 cells compared with negative control, but these inhibition effects of the silencing of DARS-AS1 or NAT10 on these biological activities of MKN45 cells were reversed by the downregulated expression of miR-330-3p. These results hinted that DARS-AS1 may promote cell proliferation, migration, and invasion through miR-330-3p/NAT10 axis.

## 4 Discussion

In the current study, we found that DARS-AS1 expression was upregulated in gastric cancer tissues and cell lines, as well as predicted shorter overall survival outcomes. Inhibition of DARS-AS1 weakened the proliferation, migration, and invasion activities of AGS and MKN45 cells. Bioinformatics prediction and dual-luciferase reporter assay results show that DARS-AS1 may sponge miR-330-3p and then modulate NAT10 in gastric cancer cells. Rescue experiments verified the effect of DARS-AS1–miR-330-3p–NAT10 interaction on cellular abilities in gastric cancer cells. These data provided a potential experimental basis for understanding the mechanism of gastric cancer.

LncRNAs are involved in the progression of various cancers and human physiological processes [21,22,23]. In gastric cancer, many lncRNA dysregulations were involved in tumor development and associated with chemosensitivity [24,25]. For instance, lncRNA ACTA2-AS1 represses malignant phenotypes of gastric cancer cells by regulating miR-378a-3p/PLCXD2 axis [26]. These findings confirmed the crucial role of lncRNAs in gastric cancer, whereas the influence of DARS-AS1 in gastric cancer remains unclear, which was upregulated using an online starBase v2.0 database [27]. Interestingly, a recent lncRNA signature study in gastric cancer did not identify DARS-AS1, which is probably because of the signature for the prediction of gastric cancer based on differential gene expression between recurrence and nonrecurrence patients [28]. The finding implied that DARS-AS1 expression may not be associated with recurrence in gastric cancer. LncRNA DARS-AS1 is a member of lncRNAs and participates in the occurrence and progression of tumors [29,30]. For instance, DARS-AS1 expression was increased in thyroid cancer tissues and related to poor prognosis, which could promote tumor cell proliferation and migration potential by regulating the expression of miR-129 [31]. DARS-AS1 could facilitate the viability and metastasis of hepatocellular carcinoma by adjusting miR-3200-5p-mediated CKAP2 [9]. Little is known about the clinical role and functional role of DARS-AS1 in gastric cancer. In this study, DARS-AS1 was also found to be overexpressed in gastric cancer tissues and predicted shorter overall survival outcomes in patients with gastric cancer, which revealed that DARS-AS1 may be correlated with prognosis for patients with gastric cancer and a potential novel prognosis predictor. Similarly, DARS-AS1 expression in thyroid cancer tissues was reported to be related to patients’ prognoses [31]. Moreover, the decreased expression of DARS-AS1 by sh-DARS-AS1 could weaken the regulation in proliferation, migration, and invasion, which was consistent with the results in other solid tumors, such as lung cancer [32] and prostate cancer [33]. These data demonstrated the crucial role of DARS-AS1 in the tumorigenesis of gastric cancer.

Many studies demonstrated that lncRNAs can act as a ceRNA competitively sponging miRNA to regulate mRNA expression in many tumors [34,35,36]. To further investigate the molecular mechanism of DARS-AS1 in gastric cancer, the DIANA online website was used to explore its potential targets. The results showed that the predicted target miR-330-3p may interact with DARS-AS1. Then, TargetScan and miRDB online databases were used to predict the downstream of miR-330-3p. NAT10 showed putative binding sites with miR-330-3p. The miR-330-3p expression could participate in the occurrence and progression of cancers [14,15,37]. Moreover, miR-330-3p functions as a tumor suppresser in gastric cancer by targeting RRRX1 [18] and MSI1 [17]. The present study explored a novel downstream target gene NAT10 of miR-330-3p. It is displayed that downregulation of DARS-AS1 increased miR-330-3p levels and overexpression of miR-330-3p inhibited the NAT10 mRNA levels, indicating a negative correlation between miR-330-3p and DARS-AS1 or NAT10. The dual-luciferase reporter assay verified the targeting relationship between either DARS-AS1 or NAT10 and miR-330-3p.

We further investigated whether miR-330-3p/NAT10 signaling contributed to DARS-AS1-induced gastric cancer cell proliferation, migration, and invasion. The rescue experiments showed that the silencing of DARS-AS1 or NAT10 alleviated proliferative potential, migration capacities, and invasion abilities, while this inhibition effect of the silencing of DARS-AS1 or NAT10 on these cellular activities was rescued by the silencing of miR-330-3p, suggesting that DARS-AS1 acts as a ceRNA through sponging miR-330-3p to enhance the expression of NAT10 in gastric cancer cells. The abnormal expression of *NAT10* has been reported in several types of cancers, which is involved in various cellular activities [38,39]. A recent study reported that NAT10 expression was increased and associated with poor prognosis, as well as had crucial effects on tumor metastasis and cell epithelial-to-mesenchymal transition (EMT) in gastric cancer [20]. Taken together, we speculated that the upregulation of DARS-AS1 weakened the viabilities and invasion of gastric cancer cells by sponging miR-330-3p to regulate NAT10 expression. The DARS-AS1/miR-330-3p/NAT10 axis interaction is involved in many tumor cellular activities, which may be an important regulatory axis in gastric cancer, whereas the detailed mechanism of DARS-AS1 in gastric cancer is still not clear and the DARS-AS1/miR-330-3p/NAT10 signaling pathway to regulate gastric cancer progression will be a study emphasis in further research.

In conclusion, this study showed that the DARS-AS1 expression was increased in gastric cancer and predicted poor survival outcomes. In addition, the silencing of DARS-AS1 and NAT10 could suppress cell behaviors by directly adjusting miR-330-3p in gastric cancer. Importantly, the DARS-AS1/miR-330-3p/NAT10 axis may help to develop novel prognosis biomarkers and treatment strategies, which may provide a new hint for comprehending the mechanism of gastric cancer progression.
